# Novel decay dynamics revealed for virus-mediated drug activation in cytomegalovirus infection

**DOI:** 10.1371/journal.ppat.1006299

**Published:** 2017-04-13

**Authors:** Jessica Rose, Vincent C. Emery, Deepali Kumar, Anders Asberg, Anders Hartmann, Alan G. Jardine, Angelo A. Bignamini, Atul Humar, Avidan U. Neumann

**Affiliations:** 1 Faculty of Life Sciences, Bar Ilan University, Ramat Gan, Israel; 2 Department of Microbial and Cellular Sciences, University of Surrey, Guildford, United Kingdom; 3 Multi Organ Transplant Program, Toronto General Hospital, Toronto, Ontario, Canada; 4 Department of Pharmaceutical Biosciences, School of Pharmacy, University of Oslo, Oslo, Norway; 5 Department of Transplant Medicine, Oslo University Hospital, Rikshospitalet, University of Oslo, Oslo, Norway; 6 Department of Medicine, University of Glasgow, Glasgow, United Kingdom; 7 School of Specialization in Hospital Pharmacy, University of Milan, Milan, Italy; 8 Institute for Theoretical Biology, Humboldt University, Berlin, Germany; 9 Institute of Environmental Medicine, Helmholtz Center Munich, UNIKA-T, Augsburg, Germany; Harvard Medical School, UNITED STATES

## Abstract

Human cytomegalovirus (CMV) infection is a substantial cause of morbidity and mortality in immunocompromised hosts and globally is one of the most important congenital infections. The nucleoside analogue ganciclovir (GCV), which requires initial phosphorylation by the viral UL97 kinase, is the mainstay for treatment. To date, CMV decay kinetics during GCV therapy have not been extensively investigated and its clinical implications not fully appreciated. We measured CMV DNA levels in the blood of 92 solid organ transplant recipients with CMV disease over the initial 21 days of ganciclovir therapy and identified four distinct decay patterns, including a new pattern exhibiting a transient viral rebound (Hump) following initial decline. Since current viral dynamics models were unable to account for this Hump profile, we developed a novel multi-level model, which includes the intracellular role of UL97 in the continued activation of ganciclovir, that successfully described all the decline patterns observed. Fitting the data allowed us to estimate ganciclovir effectiveness in vivo (mean 92%), infected cell half-life (mean 0.7 days), and other viral dynamics parameters that determine which of the four kinetic patterns will ensue. An important clinical implication of our results is that the virological efficacy of GCV operates over a broad dose range. The model also raises the possibility that GCV can drive replication to a new lower steady state but ultimately cannot fully eradicate it. This model is likely to be generalizable to other anti-CMV nucleoside analogs that require activation by viral enzymes such as UL97 or its homologues.

## Author summary

Cytomegalovirus (CMV) remains a cause of disease in individuals with weakened immune systems such as patients undergoing organ transplantation. The mainstay of treatment for CMV is ganciclovir: a CMV-specific drug that looks like a building block of the viral DNA and which requires activation by a protein contained within CMV. Understanding how patients respond to treatment can lead to a better understanding of the basic biology of CMV in the human and also help to optimize treatment regimens. In this study we observed that following treatment of CMV disease in organ transplant patients, CMV viral loads decay according to four distinct patterns. We have developed a novel mathematical model that is able to describe each of these decay patterns. The model could potentially be used to assess how different patients respond to treatment in clinical settings and also to design better drug regimens as per patient. These results have striking implications in terms of how long patients should remain on treatment and provide new insight into CMV infection in the human host.

## Introduction

Human cytomegalovirus (CMV), a member of the beta herpesvirus sub-family, has co-evolved with humans over many millennia and usually does not cause disease in the immunocompetent host [1,2]. However, in a variety of immune deficient/immature hosts including the neonate, organ transplant recipients, patients with common variable immune deficiency (CVID) and Human Immunodeficiency Virus (HIV)-infected patients, the virus can cause life-threatening pathologies [3,4]. Thus, CMV has a significant economic impact on general healthcare costs, especially in the transplant setting [5]. Despite recent encouraging results, there is no licensed vaccine against CMV[6–8]. Consequently, antiviral chemotherapy using ganciclovir (GCV) or its valine ester valganciclovir (VGCV) is currently the major clinical management tool. The drug can be given prophylactically, pre-emptively or for therapy of overt CMV syndrome and disease [9] although questions related to dosing and duration of treatment remain open [10,11].

Ganciclovir (GCV) belongs to a family of acyclic nucleoside analogues that includes aciclovir/valaciclovir (used for the clinical management of alpha herpesvirus infections such as Herpes Simplex Virus (HSV) and Varicella Zoster Virus (VZV)), which require a viral kinase (UL97 for CMV and thymidine kinase for HSV and VZV) for their initial phosphorylation step en route to the biologically active triphosphate form of the drug [12–15]. This mode of drug activation is important for the selectivity of the drug in infected cells but may also potentially result in a complex interplay between virus inhibition and drug effectiveness. Thus, understanding the interaction between CMV, expression of UL97 and GCV phosphorylation may have broader implications for herpesvirus treatment in general.

Mathematical models of viral dynamics [16] have been of immense use for our understanding of virus-drug interactions and optimization of therapy, particularly in HIV [17,18], Hepatitis C Virus (HCV) [19–22] and Hepatitis B Virus (HBV) [23,24]. However, with the exception of basic analyses of CMV kinetics during therapy [25–28], no detailed mathematical model of the dynamics of CMV, or other herpes viruses, is currently available. Consequently, we have utilized frequent viral load measurements over the first 21 days of therapy with GCV in solid organ transplant patients with CMV disease (the “VICTOR” trial [29]) to develop a comprehensive model of CMV dynamics that reveals the unique interplay between virus replication, drug activation and control of replication.

## Results

### Post-therapy CMV kinetic patterns

**Fig 1:**
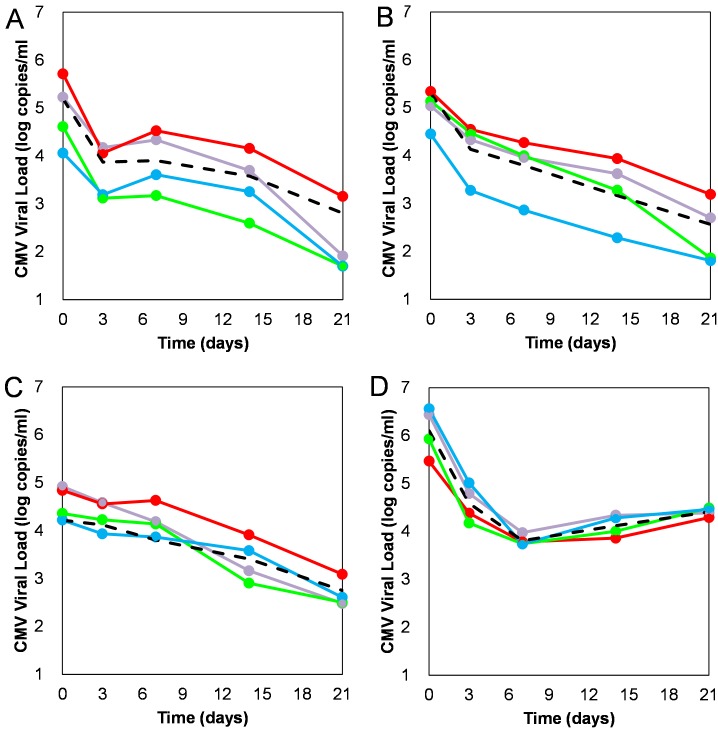
Different kinetic patterns of viral decay observed after the initiation of ganciclovir therapy (Hump (HM; panel A), Biphasic (BP; panel B), Delay (DL; panel C) and Rebound (RB; panel D). Data are shown for the mean (in black) and for four randomly-selected patients per profile (red, purple, blue and green connected points) illustrating the four profiles.

Analysis of individual viral kinetic patterns in 92 patients from the VICTOR study [29] with single CMV genotype infection (see methods for full patient information) revealed a number of kinetic patterns with distinct phases during the first month of treatment (Fig 1). In the first phase (days 0–3) most, but not all, patients showed a rapid decline in viral load. Following this decline was an intermediate phase (days 3–7 or 3–14), in which in some of the patients’ viral loads continued to decline (albeit slower), but a large fraction of patients exhibited a shoulder or transient rebound (Hump) in viral load. Lastly, almost all patients had a slow decline in CMV load on days 7–21 or 14–21. Consequently, we were able to classify all patients into 4 distinct viral kinetic patterns (see Fig 1 and Table 1; the full definition of the classification criteria is given in the methods section): Biphasic (BP, 21% of patients) where a rapid first phase decline (days 0–3) was followed by a slower second phase decline (days 3–21); Hump (HM, 60%) where an intermediate shoulder or transient rebound was observed (days 3–7, 7–14 or 3–14) between the first and second decline phases; Delay (DL, 15%) where a slow first phase decline occurred (days 0–3 or 0–7) followed by a slow, albeit faster than the first phase, second phase decline; Rebound (RB, 4%) where a rapid first phase decline was followed by a rebound between days 7–21.

**Table 1:** Summary of CMV kinetic properties per kinetic pattern group.

Viral Kinetic (VK) Property	Viral Kinetic Pattern			
(mean± StDev)	Hump (HM)	Biphasic (BP)	Delayed (DL)	Rebound (RB)
Patients per VK pattern (%)	55 (60%)	19 (21%)	14 (15%)	4 (4%)
Baseline CMV load (log ge/ml)	5.2 ± 0.7	5.3 ± 0.9	4.3 ± 0.6 *^1^	6.1 ± 0.5 *^2^
1^st^ phase slope (log ge/ml/week)	-3.2 ± 0.2	-2.3 ± 0.1	-0.5 ± 0.1	-3.5 ± 0.1
1^st^ phase decline magnitude (log ge/ml)	-1.4 ± 0.5	-1.0 ± 0.3	-0.2 ± 0.2 *^3^	-1.5 ± 0.3
Hump magnitude (log ge/ml)	+0.2 ±0.2 *^4^	-0.3 ± 0.2	-0.3 ± 0.4	-0.8 ± 0.4
2^nd^ phase slope (log ge/ml /week	-0.8 ± 0.1	-0.7 ± 0.1	-0.5 ± 0.1	+0.3 ± 0.1 *^5^
Endpoint CMV load (log ge/ml)	2.8 ± 0.8	2.6 ± 0.8	2.7 ± 0.8	4.4 ± 0.1 *^6^

*^1)^ Baseline CMV load in patients with DL is significantly (p<0.001) lower than that of HM, BP and RB.

*^2)^ Baseline CMV load in patients with RB is higher (p<0.02) than that of HM, BP and DL.

*^3)^ 1st phase decline in patients with DL is significantly (p<0.001) lower than that of HM, BP and RB (by definition).

*^4)^ Slope at days 3–7 in patients with HM is significantly (p<0.001) different than that of BP and DL (by definition).

*^5)^ 2nd phase slope at days 7–21 in patients with RB is significantly (p<0.001) different than that of HM, BP and DL (by definition).

*^6)^ Total CMV magnitude of decline at day 21 in patients with RB is higher (p = 0.008) than that of HM, BP and DL.

Such a diverse kinetic response of the same virus to the same therapy has not been observed for other viruses such as HIV and HCV. In particular, the HM pattern with a transient rebound, but nevertheless a good endpoint response, is a unique pattern not observed before in patients with consistent drug exposure. Interestingly, the mean baseline CMV load in the DL profile was significantly (p<0.001) lower than in the HM and BP profiles, while the baseline viral load in the RB group was not significantly higher (p<0.02) (Table 1 and Fig 1). The first phase decline in viral load was comparable in the BP, HM and RB groups and by definition lower in the DL patients. The second phase decline was similar in the BP, HM and DL groups. The endpoint viral loads for the HM, BP and DL profiles were similar, but not significantly higher for the RB profile as compared to the HM, BP and DL profiles (p<0.008). Moreover, we observed correlations between the baseline viral loads and the endpoint viral loads in the HM profile (R = 0.64, p<0.001), the BP profile (R = 0.74, p<0.001) and the DL profile (R = 0.63; p = 0.016), and between second phase decline and total magnitude of the viral load decline in the HM, BP and DL groups (R = 0.79, p<0.001; R = 0.82, p<0.001; R = 0.71, p = 0.004; respectively). Interestingly, we saw a trend for difference in the pattern distribution between the various CMV genotypes (Table 2), with the HM pattern more frequently observed in patients with genotype gB1 infection (69% of gB1 have HM) compared to the other genotypes (52%).

**Table 2:** Distribution of the viral kinetics patterns per CMV genotype.

Genotype	N (% of profile/genotype	HM	BP	DL	RB
gB1	42 (46%)	29 (69.0%)	6 (14.3%)	7 (16.7%)	0 (0.0%)
gB2	16 (17%)	7 (48.3%)	6 (37.5%)	2 (12.5%)	1 (6.3%)
gB3	22 (24%)	13 (59.1%)	4 (18.2%)	3 (13.6%)	2 (9.1%)
gB4	12 (13%)	6 (50.0%)	3 (25.0%)	2 (16.7%)	1 (8.3%)
**All**	**92**	**55**	**19**	**14**	**4**

### Validation of the CMV kinetic pattern classification

In order to validate our classification system, we further analyzed 89 patients with a mixed CMV genotype infection. We focused on analyzing the kinetics of the CMV gB1 genotype (the most common and most dominant genotype in our data set), which was available for 72 patients. Using the same set of classification criteria developed in the initial study, we were able to classify the viral kinetic pattern in 94% of the patients (in 4 patients the classification was not conclusive due to missing data). The HM pattern was found in 49% of the gB1 profiles in mixed-genotype infected patients, while 31% had a BP profile, 10% a DL profile and 6% a RB profile. Thus, these results validate the robustness of the classification criteria we developed previously and also show a distribution of kinetic profiles very similar to that found in the patients infected with single gB genotype infections. Interestingly, different genotypes within the same patient exhibited different kinetic patterns in 51% of the patients. We did not proceed with a full classification of the different genotypes in all patients since there are 11 different combinations of genotypes (2–4 genotypes per patient) making the sub-sets too small for a robust analysis.

### Modeling CMV viral dynamics

**Fig 2:**
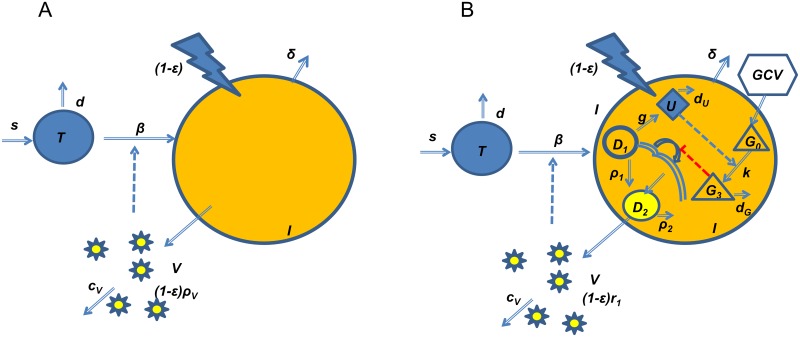
Panel A: The basic viral dynamics model, in which therapy blocks the production of virus, yields a biphasic viral decline pattern, but is not able to reproduce the complete set of kinetic patterns observed here for the effect of ganciclovir on CMV. Panel B: The novel viral dynamics model developed here for ganciclovir’s effect on CMV replication. In this model the intracellular negative feedback loop between UL97 and ganciclovir is explicitly modeled. Variable key (full description in the Methods section): *T* = target cells, *I* = infected cells, *V* = free virus, *U* = intracellular UL97 enzyme concentration, *G*_*o*_ = intracellular ganciclovir concentration, *G*_*3*_ = intracellular concentration of the active tri-phosphorylated form of ganciclovir, *D*_*1*_ = intracellular linear/circular/concatemeric forms of viral DNA, *D*_*2*_ = intracellular cleaved/packaged viral DNA, *WB* = whole blood viral load, *ε* = drug efficacy.

The standard model of viral dynamics (Fig 2a) used for analyzing HIV, HCV and HBV kinetics [16,19,23] yields a biphasic viral decline pattern if the drug is assumed to block viral production and/or secretion, or a monophasic decline if the drug is assumed to block de-novo infection. However, it cannot fit the complete data set observed here with four distinct viral decline patterns, and in particular, it is unable to model the HM pattern observed for the majority of the patients. The only way to force the standard model to give rise to a transient rebound is through a pharmacokinetic effect where drug levels are assumed to decrease at day 3 and then increase again at day 7. However, this is very unlikely in this clinical study since all patients received equal doses of drug twice a day (adjusted for renal function to maintain drug levels) throughout the 21 days of full dose medication [29,30]. Moreover, we had analyzed data for GCV dose and drug levels in the blood that were available for a subset of the patients and had not found any indication for such changes in drug levels that would account for the HM pattern. The inclusion of a transitory change in the immune response could give rise to a shoulder phase [31], if one assumes that a weaker immune response is occurring at the initiation of therapy followed by a stronger immune response after a few days or weeks of therapy. However, such a model based on changes in the immune response (affecting the loss of infected cells and or viral clearance) cannot give rise to a transitory rebound (Hump) as observed here in the majority of the patients. Evolution of resistance could explain the RB pattern but not the HM pattern, but we have verified that the HM and RB patterns were not associated with genotypic resistance to GCV (UL97 or UL54) since these patients were excluded from this analysis [32]. A number of other modifications to the standard model were tested including using a number of different infected cell populations, physiological compartments and intracellular compartments. However, none of these modifications to the model reproduced the complete set of observations, including the HM and DL patterns and the correlations mentioned above.

Thus, we developed a novel mathematical model that specifically incorporates the intracellular dynamics of the interaction between CMV and GCV (Fig 2b; see full description of the ordinary differential equation (ODE) set of equations in the methods section). A characteristic of GCV is its dependence on the UL97 viral kinase (*U*), transcribed from viral DNA (*D*_*1*_) and produced as an early/late protein whose synthesis depends on viral DNA replication, in order to be intracellularly phosphorylated from its nucleoside form (*G*_*0*_) en route to its tri-phosphate active form (*G*_*3*_). In the model, viral DNA (*D*_*1*_) replication is inhibited by the activated GCV form of ganciclovir (*G*_*3*_) with effectiveness epsilon (*ε*). The decline in DNA levels leads to a reduction in UL97 levels and accordingly, less GCV activation. As a consequence, DNA replication increases again, producing more UL97, activating more GCV, and again inhibiting DNA replication. This cycle continues until a new equilibrium, with lower CMV DNA levels than baseline, is reached between CMV DNA replication, UL97 levels and GCV activation. By incorporating these intracellular dynamics into the infected cells variable (*I*) in the standard viral kinetics model, where part of the CMV DNA (*D*_*1*_) is assembled into virions (*D*_*2*_) and exported into circulation, we obtain a specific CMV-GCV viral dynamics model. Although a small amount of GCV mono-phosphorylation occurs in the absence of UL97, we have assumed this leads to a low level of activated drug compared to a CMV infected cell and is consistent across all patients.

**Fig 3:**
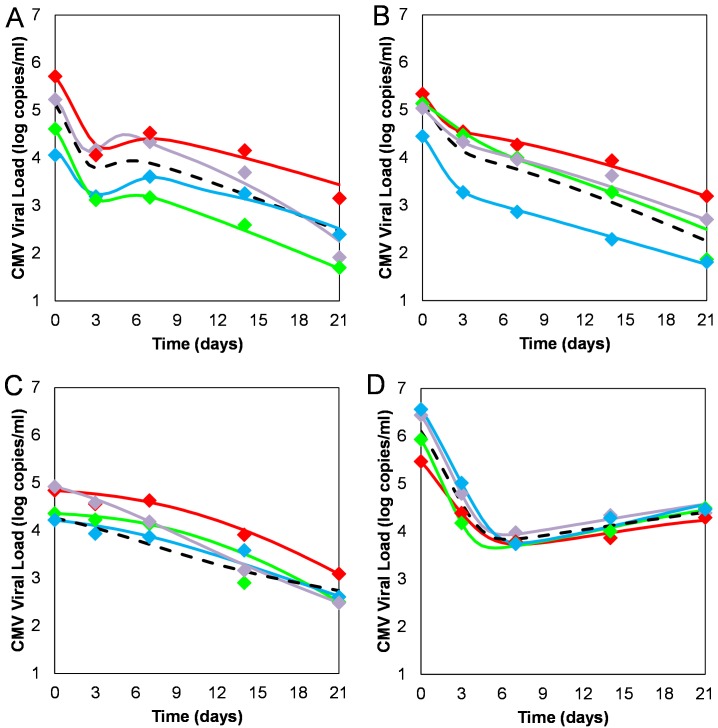
Fitting of the new GCV-CMV model to the viral decline data observed after the initiation of ganciclovir therapy per the four kinetic profiles (Hump (HM; panel A), Biphasic (BP; panel B), Delay (DL; panel C) and Rebound (RB; panel D). Data are shown for four single randomly-selected patients (red, purple, blue and green connected data points) illustrating the four profiles in tandem. The means per profile are shown in black.

Strikingly, this new model was able to account for all the viral kinetic patterns observed in the data (Fig 3, and see simulation of the kinetics of more variables of the model in S1 Fig). When the intracellular dynamics are slow, the GCV-CMV interaction has no significant impact on the blocking effectiveness of GCV, which remains approximately constant throughout therapy and hence viral decline is biphasic (BP pattern) as in the standard model. The magnitude of the first phase depicts the blocking effectiveness (*ε*) and the second phase decline slope is related to the loss rate of infected cells. However, when the dynamics of intracellular DNA, UL97 and activated GCV is fast, the blocking effectiveness is more sensitive to changes in GCV levels (Fig 3), consequently oscillations occur in the blocking effectiveness and viral DNA levels rebound more rapidly intracellularly. When this is combined with the decline of infected cells, a transient rebound in the whole blood viral load manifests (HM pattern). If the decline in infected cells is slow (a small *δ*) then a longer and more pronounced rebound is observed in the whole blood viral load time series curve (RB pattern). On the other hand, if both intracellular dynamics and intracellular DNA replication rates are slower, then UL97 levels are lower and GCV activation is reduced, with a slower effect on DNA levels, and the ensuing whole blood viral load decline is delayed (DL pattern).

### How the model explains the different CMV kinetic patterns

Of specific interest is the understanding of the differences in the model parameter values that yield each kinetic profile pattern. We found that *ε* was 8% lower on day 7 than it was on day 3 and we did not observe this difference in the other categories. This is further strong evidence that the hump occurs due to the decrease in the amount of phosphorylated GCV: the lack of available active drug resulting in a decrease in viral blocking and a subsequent increase in cell-associated viral load. We also observed a subsequent increase in *ε* by day 14 in the HM category: the value continues to decline in all other categories following day 7. We also found this pattern in *G*_*3*_: there was 68% less ganciclovir triphosphate (GCVTP; *G*_*3*_) on day 7 than on day 3 in the HM profile, and 8% more on day 14 than on day 7. This modulation in GCVTP levels does not occur in any other group. *D*_*1*_ and *D*_*2*_ decline rapidly due to the effects of *ε* and subsequently transiently increase on or around day 3 in the HM profile until both reach a new steady state.

The only qualitative or quantitative differences between the HM and BP profiles are the slopes between days 3 and 7 and the 1^st^ slope. Furthermore, since the viral load at day 0 (VL0), the endpoint viral load (VL21) and 2^nd^ slopes are almost identical between the HM and BP profiles, (Table 1) we used these two profiles as a comparative means to reveal the exact differences in parameters that yield these particular profiles. If our hypothesis is correct, then the difference in profiles should be inducible by simply modifying the intracellular parameter values associated with *U* and *G*_*3*_. The value for *d*_*u*_, the decay rate for UL97 (*U)*, is highly influential with respect to the feedback loop between *U* and *G*_*3*_ and in fact, by simply modifying this and one other parameter associated with treatment efficacy (*ε*), the HM profile can be generated. Indeed *ε* is the effectiveness of the drug at blocking viral replication and in our model; *ε* is a dynamical variable and is a function of time. This is the strength of our model: *ε* depends on the negative feedback loop between the drug and UL97 and thus depending on the particular feedback, the model is capable of simulating different viral kinetic patterns. In order to examine modifications of *ε*, we need to change the parameters that affect *ε*. The Hill parameter, *h*, is one of these parameters and represents the steepness of the Hill saturation function and defines the sensitivity of the blocking effectiveness to changes in drug level via cooperativity of drug binding. In addition to *h*, *θ* and *G*_*3*_ also affect *ε* where *θ* represents the apparent dissociation constant derived from the law of mass action and *G*_*3*_ is the ligand and is related to drug dose rather than blocking efficacy.

Interestingly, the value of *d*_*u*_ in the HM profile is higher than for the other profiles, which means that UL97 is cleared from the system more rapidly and thus less available for phosphorylating *G*_*0*_. This would set the stage for higher viral loads in the context of high treatment efficacy and perhaps temporarily offsets the ‘decay balance’ of the system inducing a transient rise in viral load. The value of the *d*_*u*_ parameter is almost completely responsible for the HM profile. The parameter *d*_*u*_ was found to be statistically-significantly different between the HM and BP profiles (p<0.001). Another parameter that differs between the HM and BP profiles is the Hill parameter, *h*, whose value partially determines the efficacy of *ε*. The value of *h* in fact dictates a change in the profile pattern. The parameter *h* was found to be significantly higher in the HM profile as compared to the BP profile (p<0.001). Ultimately, the inter-relationship between *d*_*u*_ and the treatment parameter can wholly define the HM, BP and RB kinetic profile patterns in viral load as seen by fitting their decline data (Fig 3). Interestingly, this is not case for the DL profile. Fits of individual patient data revealed that all individuals could be defined per profile *exclusively* by modifying *h* and *d*_*u*_ except for individuals with DL profile. These individual fits required more precision.

We used a parameter-fit scenario that corresponded to our biological assumptions and found that when we allowed the modification of three additional parameters: *ρ*_*1*,_ *ρ*_*2*_ and *δ*, we could fit each patient using the model. The parameter *ρ*_*1*_ is the rate at which *U* enters the system (the conversion rate from *D*_*1*_ to *D*_*2*_) and *ρ*_*2*_ is dependent on *ρ*_*1*_ (the conversion rate from *D*_*2*_ to *V*) and thus both, like *d*_*u*_, are influential with respect to the feedback loop between *U* and *G*_*3*_. Specifically, these parameters have a very pronounced impact on the 1^st^ slope. The loss rate of infected cells *δ*, albeit not influential in the feedback loop, is highly influential with respect to the 2^nd^ slope thus relevant to achieve individual fit precision, especially for the DL and RB profiles.

A complete analysis of the distribution of these 5 parameters for all patients showed a multi-modal distribution as a function of the four kinetic profiles, as shown in Supplementary Figure 2 (S2 Fig). Taking into account the differences in the numbers of patients per profile, we found that *ρ*_*1*_ was significantly different between the DL profile and all the HM, BP and RB profiles (p<0.001, p<0.001, p = 0.002; respectively). We also found no significant difference in *ρ*_*1*_ between the BP and RB profiles. *ρ*_*2*_ was significantly different between the DL profile and the HM, BP and RB profiles (p<0.001, p<0.001, p<0.001; respectively). The parameter *δ* was found to be significantly different between the RB profile and the HM and BP profiles (p<0.001, p<0.001; respectively) and between the BP and the HM, DL and RB profiles (p = 0.01, p<0.001, p<0.001, respectively). Lastly, *d*_*u*_ was found to be significantly different in the HM profile from the BP, DL and RB profiles (p<0.001, p<0.001, p<0.001; respectively). Furthermore, the values for these five parameters were not dependent on other patient or virus related co-factors. There were no significant differences in the distributions of these five parameters as function of the four CMV gB genotypes.

As part of our mathematical simulations we assessed the effects of changing the value of the decay rate for UL97 (*d*_*u*_). When we increased the value of *d*_*u*_, we saw the total amount of WB virus rise and the shape of the trajectory change until eventually there was barely a hump. This is because there was less *U* for consumption, and therefore less *G*_*3*_ and a lower value for *ε*. Thus, we observed an increase in *D*_*1*_, *D*_*2*_ and *V*. When we decreased its value, we saw the viral load decay more rapidly and the shape of the trajectory change until again, there was no appreciable hump.

Upon further examination, we noticed that the RB profile closely resembled the HM profile, albeit temporally delayed. We used the model to project the behavior of the WB viral load beyond day 21 and noticed that at day 21 the viral load in the RB profile began to decline anew. By day 90, the viral load was undetectable. Consistent with this explanation was the finding that while plasma CMV loads at day 21 were detectable (>600 genomes/ml) in 100% of RB patients it fell to 25% of RB patients by day 49. We assessed post-primary endpoint data in plasma samples for the VICTOR study patients and noted that the viral load continued to decline to undetectable levels (2.77 log genomes/ml by day 49). This has significant clinical implications in that these patients should not discontinue treatment in the face of increasing viral loads at day 21 as might be advised by a health care practitioner but instead, stay on treatment as eventually, the viral load will decline even to undetectable levels in some patients. This provides confirmation that the viral load will continue to decline after day 21 in all patients manifesting the RB profile.

### Dose-dependent viral kinetics

Lastly, we investigated what effects, if any, a lower or higher treatment dose would have on the trajectories for each kinetic profile. *G0* represents the dose of the drug and its value also partially determines the efficacy of *ε*. Thus, in order to appropriately model a dose change, rather than viral/host properties (how the drug affects blocking), we used modifications in *G0* to mimic a change in drug dose. We modified the level of *G0* to model drug efficacies that equated to *ε* at 80%, 90% and 95% for each profile (S3 Fig). According to the simulated HM, BP and RB kinetic patterns, a higher dose (in relation to the dose that yields the mean data) leads to a more substantial reduction in the nadir of the first phase decline. In the case of the HM and BP patterns, increasing the dose also results in a moderately faster second phase decline meaning that both phases are sensitive to drug dose but the first phase is far more sensitive. The DL pattern shows a dose-dependent difference in decline rates whereby the slope of the decay trajectory is progressively steeper for higher doses.

An important observation for the patients with the RB profile is that, according to the model simulations (S3 Fig), the WB ceases to increase post day 21 and actually starts to decline. Thus, according to this prediction, in a clinical setting it might be advisable for patients with the RB profile to continue treatment post day 21 in spite of the fact that it appears as though the viral load is increasing. This prediction is corroborated by the observation of low plasma viral load measurements in the VICTOR trial for the RB patients at day 49 and 84 [29].

## Discussion

Understanding CMV replication in the human host and identifying predictors for the response to therapy remains important for the clinical management of this infection in the immunocompromised host. Until now, our understanding of CMV kinetics following therapy has been limited to deploying basic models of viral dynamics. The availability of datasets from large clinical trials of antiviral medication with frequent (in particular measurement as early as day 3 of treatment) and precise (using an assay with less than 0.15 log variability) with viral load measurements is invaluable to determine viral kinetics during therapy. Using data from a clinical trial [29] comparing intravenous GCV with a valine ester pro-drug of GCV [3], we were able to both identify and validate, in 2 separate groups of patients, four distinct kinetic patterns of CMV decline during the first 21 days of therapy. While two profile patterns had been previously observed (Biphasic and Delay) [25,27,32], two new profile patterns (Hump and Rebound) were observed. The Hump (HM) profile, characterized by a rapid decline in viral load followed by a transient increase and a subsequent second phase decline, occurred in a significant proportion of the patients and was unexpected. Re-evaluation of other CMV viral load decline data from previous studies [25] indeed clearly indicates the presence of a Hump profile in some patients.

Since conventional viral dynamic models were unable to account for the four patterns observed following therapy with GCV, particularly the Hump pattern, we turned our attention to the biology of CMV-induced drug activation of GCV as a means to develop a novel multi-level model which explicitly incorporated the intracellular interplay between UL97 levels, phosphorylated anti-virally active GCV triphosphate and levels of intracellular CMV DNA. This new viral dynamic model for GCV-CMV interaction was able to account for all four kinetic patterns observed in-vivo, including the Hump profile. Although UL97 is present in the virion in small amounts, the model implies that this UL97 does not play a major role in the activation of GCV but rather plays a role in the other functions of UL97.

Our model implies that drugs such as GCV that require viral enzymes (such as UL97) for activation cannot, by themselves, eliminate viral replication from an infected cell population, arguing that an effective cell-mediated immune response is also a critical component of clearance. This has relevance in the context of recurrence of DNAemia following prophylaxis where high-risk patients who developed a T-cell response against CMV were less likely to experience late CMV infection [33] and in transplant patients treated pre-emptively where second episodes of replication are common [34].

Using model simulations, the key parameters that change one kinetic profile into another were identified as the loss rate of infected cells (*δ*) together with the parameters governing the intra-cellular feedback loop between UL97 dynamics (*d*_*u*_), DNA replication (*ρ*_*1*,_ and *ρ*_*2*_) and the GCV blocking effectiveness function (*h)*. This provides further evidence that the success of anti-herpesvirus treatment with drugs that require activation via viral proteins such as UL97 involves a complex interplay between viral replication, transcription and production of the active kinase and appropriate intracellular levels of active drug. The HM pattern that we have observed at high frequency for CMV during GCV therapy would be expected to occur with other antiviral drugs that require UL97 for activation such as valaciclovir and cyclopropavir [35]. Their efficacy at eliminating viral replication could be determined depending whether the HM pattern is observed clinically for these drugs. Studies involving patients taking anti-CMV drugs would also provide a test of our model.

Of particular interest in this work is the effect of drug dose on the viral kinetics. From our simulations, the dose of ganciclovir appears to only have a modest long-term influence on patients exhibiting the Hump (HM), Biphasic (BP) or Rebound (RB) kinetic pattern. Patients with a Delay (DL) kinetic pattern may benefit from a higher dose by exhibiting a larger decline in viral load by day 21, but unfortunately, for the majority of these patients, an undetectable viral load is hard to achieve even with higher doses. For drugs that do not require activation through a viral kinase such as the lipid ester of cidofovir (CMX001) [36] and the terminase inhibitor AIC246 (Letermovir) [37], the kinetic patterns post-therapy are likely to be predominately biphasic and not limited by the UL97 negative feedback loop. Therefore, we predict that drug efficacy will make a marked difference in time to PCR-negative status with these other drugs and thus appropriate viral kinetics based dose-ranging phase 2 studies are needed to optimize these other drugs. Such an approach would have been valuable in the context of Maribavir [38].

Of clinical relevance are the Delay and Rebound patterns. Other studies have shown that viral loads after the initiation of GCV therapy can remain static or even increase for a time after initiation of therapy [39,40] and in one study this has been shown to be associated with rapid replication kinetics prior to therapy [41]. In our analysis, patients with the Delay pattern tended to have lower baseline viral loads, possibly indicating that these patients had intrinsically low CMV replication rates giving rise to low availability of UL97, thus limiting the quantity of intracellular active GCV and giving rise to a delayed viral decline.

Many patients are deemed as non-responsive to GCV therapy if by day 21 the viral load is not declining or has not declined to an undetectable level [29]. In light of this, drug resistance may be suspected and in some patients switching to other drugs such as Foscarnet or Cidofovir would be considered. However, our mathematical model predicts that at least in some of these patients, specifically patients with no drug resistance mutations, the observed Rebound (RB) group is a ‘slowed down’ version of the Hump kinetic pattern. Thus, maintaining GCV therapy at full dose in these RB patients may be warranted since CMV loads could start declining again, perhaps even to undetectable levels over the following 28 days. This prediction is supported by the observation in the VICTOR study that patients who had not cleared CMV from their blood by day 21 and who were in the RB kinetic profile became PCR-negative in plasma by day 49 or 84 [29].

An important limitation of our study is the lack of pre-treatment viral load data and hence it is not possible to verify that viremia was at steady state at the initiation of therapy, as assumed in the model. Indeed it has been previously shown by us [41] in stem cell transplant patients that in some patients, therapy is initiated when CMV is rapidly increasing thus potentially altering the kinetic pattern after initiation of therapy. However, this occurred in patients being treated pre-emptively with viral loads less than 10,000 genomes per ml of blood, while, in contrast, the patients in the VICTOR study had much higher viral loads when ganciclovir treatment was initiated. Moreover, the stem cell transplant patients with a pre-treatment rapid viral load increase showed a continued increase at the beginning of therapy [41], while here we did not observe any patient in the present study with an increase in viral load between days 0 to 3.

At present, our modeling effort focused on patients with mono-genotypic infection (as judged by glycoprotein B typing) however, we have validated our kinetic pattern classification (and model) in patients with multi-genotypic infections [42,43] and again, observed all 4 viral decay patterns. Unfortunately, because of the distribution of different combinations of gB genotypes in the mixed-genotype infected patients, the sub-sets were too small for us to undertake a full analysis.

In conclusion, the central tenet of our CMV-GCV model is the negative feedback loop between CMV replication and GCV activation, which is mediated through the dynamics of the UL97 kinase. This allows a more accurate description of CMV kinetics following antiviral therapy. We expect these insights to contribute to understanding and optimizing the effective clinical management of CMV with existing and new antiviral compounds in advanced clinical development.

## Materials and methods

### Patients and viral load measurement

The VICTOR study included patients who were solid organ recipients with cytomegalovirus (CMV) disease at the time of enrolment. It was a randomized, open-label, parallel-group, active drug-controlled, multi-center non-inferiority trial in adult solid organ transplant recipients with CMV disease (ClinicalTrials.gov NCT00431353) and was conducted in accordance with the Declaration of Helsinki, good clinical practice guidelines and applicable local regulatory requirements as previously reported [29]. This 2-arm study evaluated the efficacy and safety of 2 forms of ganciclovir (GCV): oral valganciclovir (Valcyte) compared with intravenous ganciclovir for the treatment of CMV disease. Eligible patients were randomized to receive either 1) valganciclovir 900 mg po bid or 2) Ganciclovir 5mg/kg iv bid for 21 days, and then both arms continued with maintenance valganciclovir [dose 900 mg/day until day 49] with all dosages adjusted for renal function [29]. For the purposes of our kinetic analysis, we included only the 113 patients in whom a single CMV gB genotype infection was present. As a validation step we tested our classification criteria on an additional 89 patients with a mixed CMV gB genotype infection. Patients with any genotypic mutations possibly associated with resistance to GCV, as measured by a nested PCR assay covering (UL97 and UL54) all known resistance mutations to GCV [44], tested at all visits during the study, were excluded from the analysis.

Whole blood (WB) CMV viral loads were measured at days 0, 3, 7, 14 and 21 by a CMV genotype specific real-time PCR assay as previously described [45]. This in-house assay was validated to have better inter-assay variability (0.1–0.15 log) than commercial assays for CMV quantification and a high sensitivity (LLD = 50 copies/ml, LLQ = 500 copies/ml) [45]. Patients that were missing viral load data between days 0 and 21 were omitted from the analysis. Consequently, we analyzed viral kinetics in a total of 92 patients with a single genotype infection in the initial analysis, and gB1 viral kinetics in an additional 72 patients a mixed-genotype infection in the validation step. There was no difference in viral kinetics between the 2 study arms and thus we analyzed the cohort as a whole [29].

### Ethical statement

The sub-study described in this paper was approved by the Norwegian Ethics Committee, Section for Health Region South-East (IRB number: S-04011). All data was anonymized prior to analysis. All patients had given written informed consent.

### Viral kinetics pattern classification

The four kinetic profiles were assigned based upon the viral kinetic pattern during the first 21 days of therapy. A patient was classified as having a Biphasic (BP) profile if the first phase (days 0–3) showed a rapid decline, larger than 0.5 log, and then a second phase (days 3–21) with a slower continuous decline. A Hump (HM) profile was defined by a first phase (days 0–3) with a rapid decline, larger than 0.5 log, followed by an intermediate shoulder or hump phase (at days 3–7, 7–14 or 3–14) with no viral decline (less than 0.15 log decline, where 0.15 log is the inter-assay variability of the assay used to quantify CMV load) or with a viral load increase, but nevertheless a later second phase decline (days 7–21 or 14–21). A Rebound (RB) profile was defined by a rapid viral load decline in the first phase (days 0–3), larger than 0.5 log, followed by a continuous increase in viral load at days 3–21 or 7–21, instead of a second phase decline. A Delay (DL) profile was defined by a slow first phase decline (days 0–3) of less than 0.5 logs, followed by a decline at days 3–21. It should be noted that we made no *a priori* hypothesis related to the number or nature of the kinetic patterns, but rather selected the criteria that minimized the number of kinetic patterns.

### Statistical analysis

Statistical analyses were done using *SPSS*. Correlations were tested for statistical significance using the non-parametric Spearman test. The Fischer exact test was used to test statistical significance in differences of proportions between groups. The non-parametric Mann-Whitney test was used to determine if differences in distribution of parameters between groups of patients were statistically significant. The significance threshold was set at 0.05 except where multiple comparisons were performed in which the threshold was reduced accordingly. In the text, we have only highlighted significant differences where the p-values fall below a 0.0017 threshold.

### Viral dynamics model of the GCV-CMV interaction

CMV transcription occurs in three stages: early, intermediate and late with DNA replication occurring after early transcription and via the production of a concatemeric structure that is then cleaved and packaged into new virions [3]. GCV triphosphate (GCVTP) interrupts this process by inhibiting viral DNA replication. We assume that this occurs at the lengthening stage: the drug inhibits lengthening of the viral DNA so that the precursor DNA for packaging is ultimately not produced. Studies have shown that GCV triphosphate levels are at least 100-fold greater in CMV-infected cells than in non-infected cells [13]. In order for GCV in the cells to be transformed into GCVTP it has to be initially phosphorylated by the CMV kinase UL97. Since UL97 is transcribed as an early-late gene, which likely requires DNA replication for maximum expression, we hypothesized that UL97 levels change according to the change in intracellular replication levels. Therefore, changing replication levels would result in changes of GCVTP levels, which ultimately would change the blocking efficacy of ganciclovir.

Thus, based upon the above assumptions, we have developed a viral dynamic model that combines the intracellular interaction between GCV and UL97 together with the blocking effect of GCV on CMV. Briefly, *G*_*0*_ is the intracellular GCV drug concentration. *D*_*1*_ represents the linear/circular/concatemeric forms of viral DNA, and *D*_*2*_ represents the cleaved and packaged viral DNA. UL97 phosphorylates GCV (*G*_*0*_) to produce a mono-phosphorylated form of the drug (*G*_*1*_) which is rapidly metabolized to the active tri-phosphorylated form of the drug (*G*_*3*_) by cellular kinases. This active form of the drug then acts primarily on the replication of *D*_*1*_ to inhibit the prolongation of the linear DNA, formation of circular DNA and subsequent packaging of DNA for new virions. Since *D*_*1*_ is assumed to be the template for UL97 transcripts and hence the UL97 protein, this produces a negative-feedback loop causing the eventual reduction in both *D*_*1*_ and subsequently UL97 (Fig 2b).

In the absence of adequate quantities of UL97, the drug, which continues to enter the cell (as patients are undergoing continued dosing twice a day) does not get phosphorylated efficiently and so drug efficacy is reduced. This means that the viral content in the cell in the form of *D*_*1*_ and *D*_*2*_ eventually begins to rise, peak and subsequently plateau at new steady state levels. In addition, since *V* originates from *D*_*2*_, *V* also declines and then rises albeit, more slowly. Once UL97 is restored to large enough quantities due to continued viral replication, the drug is again phosphorylated, can act as an inhibitor of DNA synthesis, and the cycle continues. This could manifest as a transient rise in intracellular (cell-associated) DNA content and gives rise to oscillatory behavior.

The differential equations that follow from above assumptions (see schematic representation in Fig 2b) are as follows.

dT/dt = s – dT−βTV

dI/dt = βTV−δI

$$dU/dt\;=\;gD_{1}-d_{u}U$$

$$dG_{3}/dt=\;kUG_{0}-d_{G}G_{3}$$

$$dD_{1}/dt\;=\;\left(1-\varepsilon \right)r_{1}-\rho_{1}D_{1}$$

$$dD_{2}/dt\;=\;\rho_{1}D_{1}-\rho_{2}D_{2}$$

$$dV/dt\;=\;P_{frac}\rho_{2}D_{2}I-c_{V}V$$

where *P*_*frac*_ = *q*.

The model contains seven variables that represent: uninfected cells (*T)*, infected cells (*I)*, intracellular UL97 enzyme (*U)*, the intracellular triphosphate form of GCV (*G*_*3*_), the linear form of the viral DNA which becomes elongated for eventual cleavage into component viral parts, (*D*_*1*_), assembled capsid forms in which the scaffolding has been removed and replaced with viral DNA (*D*_*2*_), and consequently released to the circulation as free virus (*V*). Uninfected cells are produced at a rate *s*, die at a rate *d*, and become infected with rate constant *β*. Infected cells are lost at a rate *δ*. *UL97* is produced at rate *g* from the linear DNA form (D_1_) and is lost at rate *d*_*u*_. *G*_*3*_ is produces at rate *k* as a first order linear interaction of UL97 and the non-phosphorylated form of GCV (*G*_*0*_) and lost at rate *d*_*G*_. The linear DNA form (*D*_*1*_) replicates at rate *r*_*1*_ and is transformed at rate *ρ*_*1*_ to produce *D*_*2*_ that is lost at rate *ρ*_*2*_. A fraction *P*_*frac*_ (*q*) (/ml) of this cleaved virus (*D*_*2*_) becomes free virus with a clearance rate of *c*_*V*_. The variables in the model represent populations expressed as concentration per milliliter of blood and specifically, the units for cell populations are cells/ml while viral population units are virions/ml. Both the viral enzyme and the drug population units are molecules/cell/ml. It is assumed that mixing in each compartment is instantaneous—that is, intracellular and extracellular diffusion are assumed to be fast on the scale of an individual cell. Parameter rate constants are summarized follows (Table 3).

**Table 3:** Units of variables and parameters.

Variable	Units	Parameters	Units
*T*	cells/ml	*s*	cells/ml/day
*I*	cells/ml	*β*	ml/virion/day
*U*	molecules/cell/ml	*D*	1/day
*G*_*3*_	molecules/cell/ml	*δ*	1/day
*D*_*1*_	virions/cell/ml	*g*	molecules/virion/day
*D*_*2*_	virions/cell/ml	*d*_*u*_	1/day
*V*	virions/ml	*k*	(ml/molecule)*(cell/day)
		*d*_*g*_	1/day
		*R*_*1*_	virions/cell/ml/day
		*ρ*_*1*_	1/day
		*ρ*_*2*_	1/day
		*c*_*V*_	1/day

To represent the antiviral effect of GCV we introduce a parameter, *ε*, to block the production rate of *D*_*1*_ where the blocking effectiveness depends on the availability of GCV by:

$$\varepsilon =G_{3}{}^{h}/\left(\theta^{h}+G_{3}{}^{h}\right).$$

This equation utilizes the Hill function: the Hill coefficient, *h*, represents the steepness of the Hill saturation function and defines the sensitivity of the blocking effectiveness to changes in drug level via cooperativity of drug binding.

The total whole blood viral load is then calculated as:

$$\text{WB }=\;(D_{1}+\;D_{2})I+V$$

and this variable is fitted to the measured viral load.

All modeling was performed with the Berkeley Madonna package and fitting performed using the Runge-Kutta4 method.

### Data fitting

Considering that our model is novel and has 7 variables (only one of which is measurable) and 15 parameters which are very difficult to measure experimentally in vivo (and outside of the scope of this particular work), we explored different parameter sets or landscapes in order to identify which of the parameters, or sets of parameters, were the most influential in giving rise to one kinetic profile or another. We systematically examined the sensitivity of each parameter while keeping the rest constant to primarily assess the effect that each had on the system. This was done by data fitting numerically using the Runge-Kutta iterative method. We also investigated potential relationships between the parameters themselves. Once we had established which parameters were most sensitive, we sought to minimize the number of free-fit parameters in order to be more robust in our analysis. We subsequently investigated which parameters must remain constant in order to obtain all 4 kinetic profiles. We set these selected parameters at constant values that were based on a quasi steady state analysis and available biological information with previously generated estimates [46]. We used the relationships observed between the parameters from the quasi steady state analysis to characterize each parameter. By replacing each parameter with a scaled version, we figured out if each represented a combination of other parameters or non-observed variables. To try to understand exactly why we see the unique profiles, we examined the effects of changing each parameter, alone and in tandem with others for each profile. We found that some parameters dictate the profile patterns while others simply promote them. Consequently, five parameters were identified that produce the differences between one kinetic profile and another: *ρ*_*1*,_ *ρ*_*2*,_ *h*, *δ* and *d*_*u*_. The values of the other parameters were fixed in the simulations and in the data fitting to: *s* = 10^6^; *d* = 0.1; *k* = 10^5^; *g* = 0.01; *G*_*0*_ = 0.06; *d*_*G*_ = 1; *θ* = 10^3^; *r*_*1*_ = 1000, *c* = 0.26; *P*_*frac*_ = 0.09. We tested a large range of other values for the fixed parameters and the qualitative results reported here, in relation to the free parameter values, did not change.

## Supporting information

S1 Fig Simulations of the GCV-CMV model showing the kinetics of cellular level variables: Whole blood viral load (*WB*, black curves) and infected cells (*I*, green dashed curves), and intracellular level variables: UL97 kinase (*U*, blue curves), active tri-phosphorylated form of ganciclovir (*G*_*3*_, red dots) and linear/circular/concatemeric forms of viral DNA (*D*_*1*_, blue dashed curves). Each simulation is fitted to viral load data (blue triangles) from a representative patient from each kinetic profile, thus depicting the differences in the behavior of these variables between the four kinetic profiles (HM (panel A), BP (panel B), DL (panel C) and RB (panel D)).
(TIF)

S2 Fig A histogram of the distribution of the five parameters (*ρ*_*1*_, *ρ*_*2*_, *h*, *δ* and *d*_*u*_) that were free to vary in the fitting of the viral kinetic data of all patients across the different kinetic profiles (HM (panel A), BP (panel B), DL (panel C) and RB (panel D)). (TIF)

S3 Fig Effect of dose changes in GCV is simulated for each kinetic profile (HM, BP, DL and RB) by changes in the drug effectiveness, *ε*, in blocking CMV DNA replication. *G0* was modified to mimic a change in drug dose that mimicked drug efficacies that equated to *ε* at 80% (black solid line), 90% (mean—dashed black line) and 95% (dotted black line) for each profile. The mean data is also shown for each profile. The limit of detection (LOD) of the viral load (400 copies/ml) is shown by the horizontal green line.
(TIF)

## References

[1] McGeoch DJ, Dolan A, Ralph AC. Toward a comprehensive phylogeny for mammalian and avian herpesviruses. J Virol. 2000;74(22):10401–6. 11044084 10.1128/jvi.74.22.10401-10406.2000 PMC110914

[2] Engel P, Angulo A. Viral immunomodulatory proteins: usurping host genes as a survival strategy. Adv Exp Med Biol. 2012;738:256–76. 10.1007/978-1-4614-1680-7_15 22399384

[3] Griffiths PD. Chapter 8 Cytomegalovirus in Principles and Practices of Clinical Virology (6th edition) (ed. Zuckerman A. J., Banatvala J. E., Griffiths P.D., Schoub B., Mortimer P.)2009 161–97 p.

[4] Fishman JA, Emery V, Freeman R, Pascual M, Rostaing L, Schlitt HJ, et al Cytomegalovirus in transplantation—challenging the status quo. Clin Transplant. 2007;21(2):149–58. 10.1111/j.1399-0012.2006.00618.x 17425738

[5] Paya CV. Economic impact of cytomegalovirus in solid organ transplantation. Transpl Infect Dis. 2001;3 Suppl 2:14–9. 11926744 10.1034/j.1399-3062.2001.00003.x

[6] Pass RF, Zhang C, Evans A, Simpson T, Andrews W, Huang ML, et al Vaccine prevention of maternal cytomegalovirus infection. N Engl J Med. 2009;360(12):1191–9. 10.1056/NEJMoa0804749 19297572 PMC2753425

[7] Griffiths PD, Stanton A, McCarrell E, Smith C, Osman M, Harber M, et al Cytomegalovirus glycoprotein-B vaccine with MF59 adjuvant in transplant recipients: a phase 2 randomised placebo-controlled trial. Lancet. 2011;377(9773):1256–63. 10.1016/S0140-6736(11)60136-0 21481708 PMC3075549

[8] Kharfan-Dabaja MA, Boeckh M, Wilck MB, Langston AA, Chu AH, Wloch MK, et al A novel therapeutic cytomegalovirus DNA vaccine in allogeneic haemopoietic stem-cell transplantation: a randomised, double-blind, placebo-controlled, phase 2 trial. Lancet Infect Dis. 2012;12(4):290–9. 10.1016/S1473-3099(11)70344-9 22237175

[9] Biron KK. Antiviral drugs for cytomegalovirus diseases. Antiviral Res. 2006;71(2–3):154–63. 10.1016/j.antiviral.2006.05.002 16765457

[10] Hodson EM, Ladhani M, Webster AC, Strippoli GF, Craig JC. Antiviral medications for preventing cytomegalovirus disease in solid organ transplant recipients. Cochrane Database Syst Rev. 2013(2):CD003774 10.1002/14651858.CD003774.pub4 23450543

[11] Baldanti F, Lilleri D, Gerna G. Human cytomegalovirus load measurement and its applications for pre-emptive therapy in patients undergoing hematopoietic stem cell transplantation. Hematol Oncol. 2008;26(3):123–30. 10.1002/hon.856 18386849

[12] Littler E, Stuart AD, Chee MS. Human cytomegalovirus UL97 open reading frame encodes a protein that phosphorylates the antiviral nucleoside analogue ganciclovir. Nature. 1992;358(6382):160–2. 10.1038/358160a0 1319559

[13] Sullivan V, Talarico CL, Stanat SC, Davis M, Coen DM, Biron KK. A protein kinase homologue controls phosphorylation of ganciclovir in human cytomegalovirus-infected cells. Nature. 1992;358(6382):162–4. 10.1038/358162a0 1319560

[14] Elion GB. Mechanism of action and selectivity of acyclovir. Am J Med. 1982;73(1A):7–13. 6285736 10.1016/0002-9343(82)90055-9

[15] Roberts GB, Fyfe JA, McKee SA, Rahim SG, Daluge SM, Almond MR, et al Varicella-zoster virus thymidine kinase. Characterization and substrate specificity. Biochem Pharmacol. 1993;46(12):2209–18. 8274154 10.1016/0006-2952(93)90611-y

[16] Nowak M.A, May R.M. Virus Dynamics: Oxford University Press; 2000.

[17] Perelson AS, Neumann AU, Markowitz M, Leonard JM, Ho DD. HIV-1 dynamics in vivo: virion clearance rate, infected cell life-span, and viral generation time. Science. 1996;271(5255):1582–6. 8599114 10.1126/science.271.5255.1582

[18] Bonhoeffer S, May RM, Shaw GM, Nowak MA. Virus dynamics and drug therapy. Proc Natl Acad Sci U S A. 1997;94(13):6971–6. 9192676 10.1073/pnas.94.13.6971 PMC21269

[19] Guedj J, Perelson AS. Second-phase hepatitis C virus RNA decline during telaprevir-based therapy increases with drug effectiveness: implications for treatment duration. Hepatology. 2011;53(6):1801–8. 10.1002/hep.24272 21384401 PMC3103645

[20] Neumann AU, Lam NP, Dahari H, Gretch DR, Wiley TE, Layden TJ, et al Hepatitis C viral dynamics in vivo and the antiviral efficacy of interferon-alpha therapy. Science. 1998;282(5386):103–7. 9756471 10.1126/science.282.5386.103

[21] Dahari H, Major M, Zhang X, Mihalik K, Rice CM, Perelson AS, et al Mathematical modeling of primary hepatitis C infection: noncytolytic clearance and early blockage of virion production. Gastroenterology. 2005;128(4):1056–66. 15825086 10.1053/j.gastro.2005.01.049

[22] Zeuzem S, Pawlotsky JM, Lukasiewicz E, von Wagner M, Goulis I, Lurie Y, et al International, multicenter, randomized, controlled study comparing dynamically individualized versus standard treatment in patients with chronic hepatitis C. J Hepatol. 2005;43(2):250–7. 16082736 10.1016/j.jhep.2005.05.016

[23] Nowak MA, Bonhoeffer S, Hill AM, Boehme R, Thomas HC, McDade H. Viral dynamics in hepatitis B virus infection. Proc Natl Acad Sci U S A. 1996;93(9):4398–402. 8633078 10.1073/pnas.93.9.4398 PMC39549

[24] Whalley SA, Murray JM, Brown D, Webster GJ, Emery VC, Dusheiko GM, et al Kinetics of acute hepatitis B virus infection in humans. J Exp Med. 2001;193(7):847–54. 11283157 10.1084/jem.193.7.847 PMC2193367

[25] Emery VC, Cope AV, Bowen EF, Gor D, Griffiths PD. The dynamics of human cytomegalovirus replication in vivo. J Exp Med. 1999;190(2):177–82. 10432281 10.1084/jem.190.2.177 PMC2195570

[26] Emery VC, Griffiths PD. Prediction of cytomegalovirus load and resistance patterns after antiviral chemotherapy. Proc Natl Acad Sci U S A. 2000;97(14):8039–44. 10.1073/pnas.140123497 10859361 PMC16666

[27] Wolf DG, Shimoni A, Resnick IB, Stamminger T, Neumann AU, Chou S, et al Human cytomegalovirus kinetics following institution of artesunate after hematopoietic stem cell transplantation. Antiviral Res. 2011;90(3):183–6. 10.1016/j.antiviral.2011.03.184 21443904 PMC3253856

[28] Emery VC, Hassan-Walker AF, Burroughs AK, Griffiths PD. Human cytomegalovirus (HCMV) replication dynamics in HCMV-naive and -experienced immunocompromised hosts. J Infect Dis. 2002;185(12):1723–8. 10.1086/340653 12085317

[29] Asberg A, Humar A, Rollag H, Jardine AG, Mouas H, Pescovitz MD, et al Oral valganciclovir is noninferior to intravenous ganciclovir for the treatment of cytomegalovirus disease in solid organ transplant recipients. Am J Transplant. 2007;7(9):2106–13. 10.1111/j.1600-6143.2007.01910.x 17640310

[30] Perrottet N, Manuel O, Lamoth F, Venetz JP, Sahli R, Decosterd LA, et al Variable viral clearance despite adequate ganciclovir plasma levels during valganciclovir treatment for cytomegalovirus disease in D+/R- transplant recipients. BMC Infect Dis. 2010;10:2 10.1186/1471-2334-10-2 20053269 PMC2820479

[31] Layden JE, Layden TJ, Reddy KR, Levy-Drummer RS, Poulakos J, Neumann AU. First phase viral kinetic parameters as predictors of treatment response and their influence on the second phase viral decline. J Viral Hepat. 2002;9(5):340–5. 12225328 10.1046/j.1365-2893.2002.00377.x

[32] Emery VC, Manuel O, Asberg A, Pang X, Kumar D, Hartmann A, et al Differential decay kinetics of human cytomegalovirus glycoprotein B genotypes following antiviral chemotherapy. J Clin Virol. 2012;54(1):56–60. 10.1016/j.jcv.2012.01.015 22410132 PMC3328767

[33] Kumar D, Chernenko S, Moussa G, Cobos I, Manuel O, Preiksaitis J, et al Cell-mediated immunity to predict cytomegalovirus disease in high-risk solid organ transplant recipients. Am J Transplant. 2009;9(5):1214–22. 10.1111/j.1600-6143.2009.02618.x 19422346

[34] Atabani SF, Smith C, Atkinson C, Aldridge RW, Rodriguez-Peralvarez M, Rolando N, et al Cytomegalovirus replication kinetics in solid organ transplant recipients managed by preemptive therapy. Am J Transplant. 2012;12(9):2457–64. 10.1111/j.1600-6143.2012.04087.x 22594993 PMC3510308

[35] Kern ER, Bidanset DJ, Hartline CB, Yan Z, Zemlicka J, Quenelle DC. Oral activity of a methylenecyclopropane analog, cyclopropavir, in animal models for cytomegalovirus infections. Antimicrob Agents Chemother. 2004;48(12):4745–53. 10.1128/AAC.48.12.4745-4753.2004 15561852 PMC529216

[36] Marty FM, Winston DJ, Rowley SD, Vance E, Papanicolaou GA, Mullane KM, et al CMX001 to prevent cytomegalovirus disease in hematopoietic-cell transplantation. N Engl J Med. 2013;369(13):1227–36. 10.1056/NEJMoa1303688 24066743

[37] Stoelben S, Arns W, Renders L, Hummel J, Muhlfeld A, Stangl M, et al Preemptive treatment of Cytomegalovirus infection in kidney transplant recipients with letermovir: results of a Phase 2a study. Transpl Int. 2014;27(1):77–86. 10.1111/tri.12225 24164420

[38] Winston DJ, Saliba F, Blumberg E, Abouljoud M, Garcia-Diaz JB, Goss JA, et al Efficacy and safety of maribavir dosed at 100 mg orally twice daily for the prevention of cytomegalovirus disease in liver transplant recipients: a randomized, double-blind, multicenter controlled trial. Am J Transplant. 2012;12(11):3021–30. 10.1111/j.1600-6143.2012.04231.x 22947426

[39] Nichols WG, Corey L, Gooley T, Drew WL, Miner R, Huang M, et al Rising pp65 antigenemia during preemptive anticytomegalovirus therapy after allogeneic hematopoietic stem cell transplantation: risk factors, correlation with DNA load, and outcomes. Blood. 2001;97(4):867–74. 11159510 10.1182/blood.v97.4.867

[40] Gerna G, Lilleri D, Zecca M, Alessandrino EP, Baldanti F, Revello MG, et al Rising antigenemia levels may be misleading in pre-emptive therapy of human cytomegalovirus infection in allogeneic hematopoietic stem cell transplant recipients. Haematologica. 2005;90(4):526–33. 15820949

[41] Buyck HC, Griffiths PD, Emery VC. Human cytomegalovirus (HCMV) replication kinetics in stem cell transplant recipients following anti-HCMV therapy. J Clin Virol. 2010;49(1):32–6. 10.1016/j.jcv.2010.06.018 20667769

[42] Ross SA, Novak Z, Pati S, Patro RK, Blumenthal J, Danthuluri VR, et al Mixed infection and strain diversity in congenital cytomegalovirus infection. J Infect Dis. 2011;204(7):1003–7. 10.1093/infdis/jir457 21881114 PMC3164425

[43] Pati SK, Pinninti S, Novak Z, Chowdhury N, Patro RK, Fowler K, et al Genotypic diversity and mixed infection in newborn disease and hearing loss in congenital cytomegalovirus infection. Pediatr Infect Dis J. 2013;32(10):1050–4. 10.1097/INF.0b013e31829bb0b9 23694837 PMC3785554

[44] Boivin G, Goyette N, Gilbert C, Roberts N, Macey K, Paya C, et al Absence of cytomegalovirus-resistance mutations after valganciclovir prophylaxis, in a prospective multicenter study of solid-organ transplant recipients. J Infect Dis. 2004;189(9):1615–8. 10.1086/382753 15116297

[45] Pang X, Humar A, Preiksaitis JK. Concurrent genotyping and quantitation of cytomegalovirus gB genotypes in solid-organ-transplant recipients by use of a real-time PCR assay. J Clin Microbiol. 2008;46(12):4004–10. 10.1128/JCM.01341-08 18971365 PMC2593284

[46] Kepler GM, Banks HT, Davidian M, Rosenberg ES. A Model for HCMV Infection in Immunosuppressed Patients. Math Comput Model. 2009;49(7–8):1653–63. 10.1016/j.mcm.2008.06.003 20161307 PMC2699305

