# Correction: Novel decay dynamics revealed for virus-mediated drug activation in cytomegalovirus infection

**DOI:** 10.1371/journal.ppat.1006386

**Published:** 2017-05-10

**Authors:** Jessica Rose, Vincent C. Emery, Deepali Kumar, Anders Asberg, Anders Hartmann, Alan G. Jardine, Angelo A. Bignamini, Atul Humar, Avidan U. Neumann

The images for S2 and S3 Figs are incorrectly switched. The image that appears as S2 Fig should be S3 Fig, and the image that appears as S3 Fig should be S2 Fig. The figure captions appear in the correct order.
