# Supplementary figures and images for: Novel decay dynamics revealed for virus-mediated drug activation in cytomegalovirus infection
Source: PLoS Pathog. 2017 Apr 13;13(4):e1006299. doi: 10.1371/journal.ppat.1006299 (PMC5391089; doi:10.1371/journal.ppat.1006299)

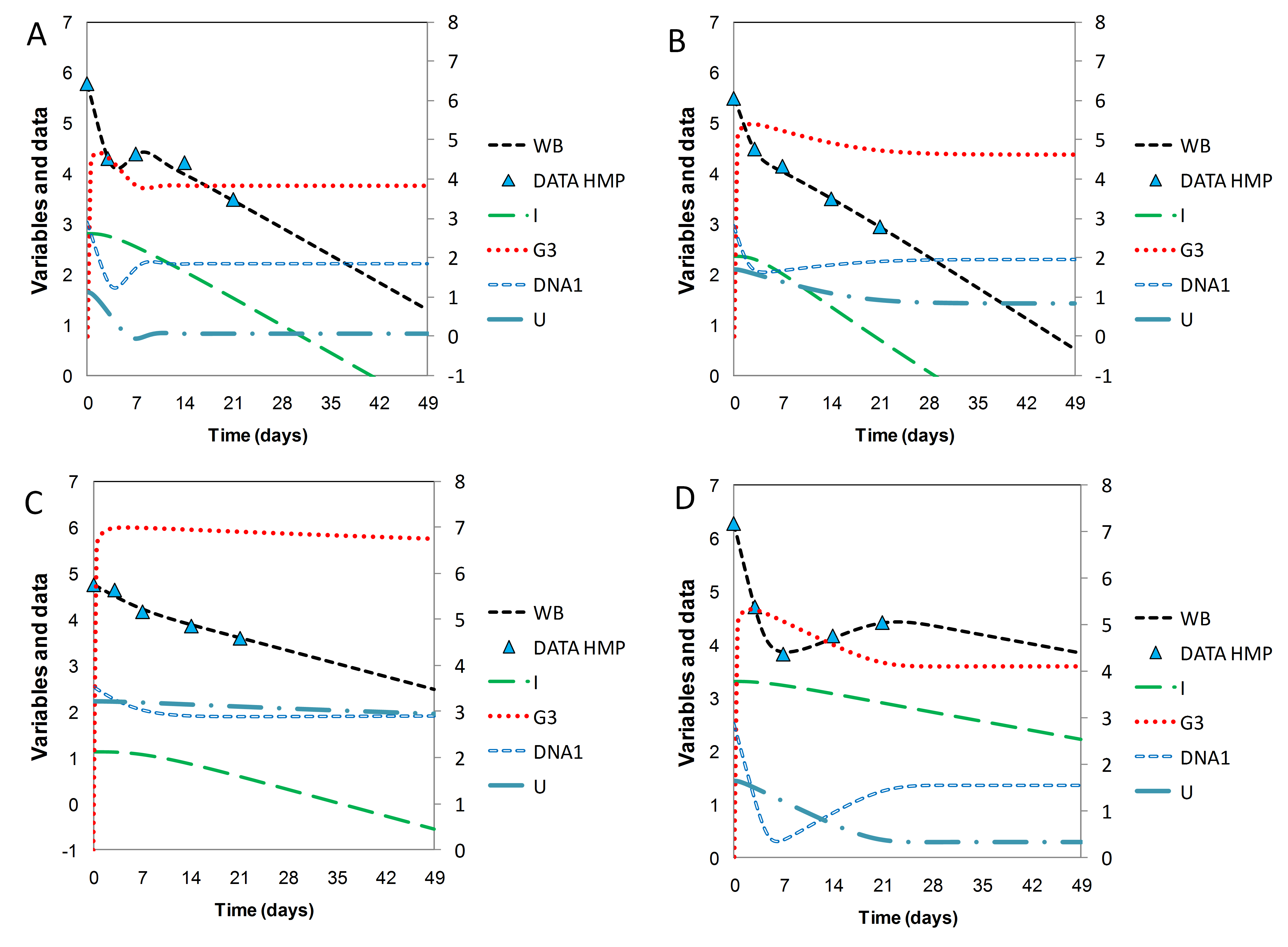

Supplement: S1 Fig — Each simulation is fitted to viral load data (blue triangles) from a representative patient from each kinetic profile, thus depicting the differences in the behavior of these variables between the four kinetic profiles (HM (panel A), BP (panel B), DL (panel C) and RB (panel D)). (TIF) [file ppat.1006299.s001.tif]

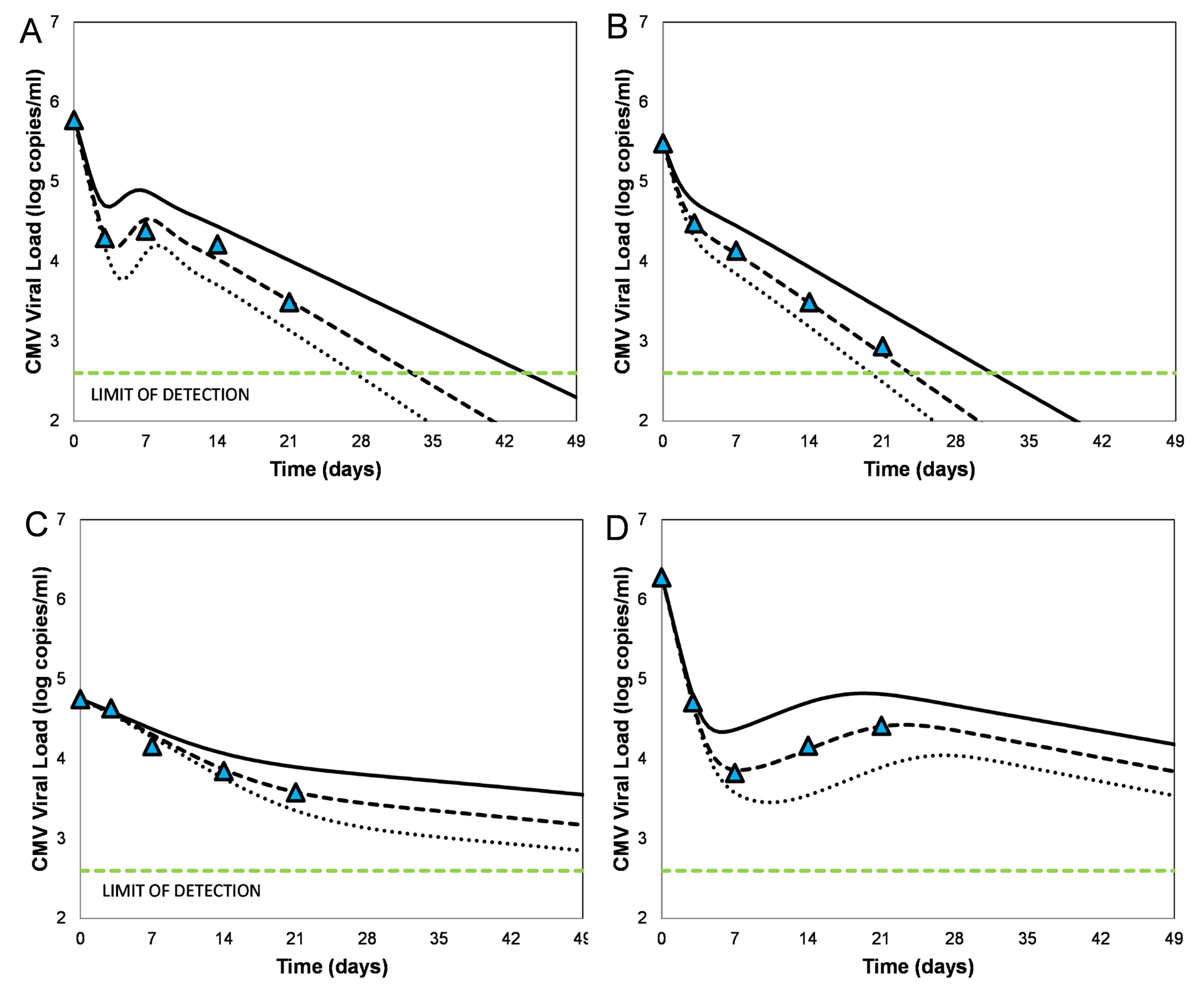

Supplement: S2 Fig — (TIF) [file ppat.1006299.s002.tif]

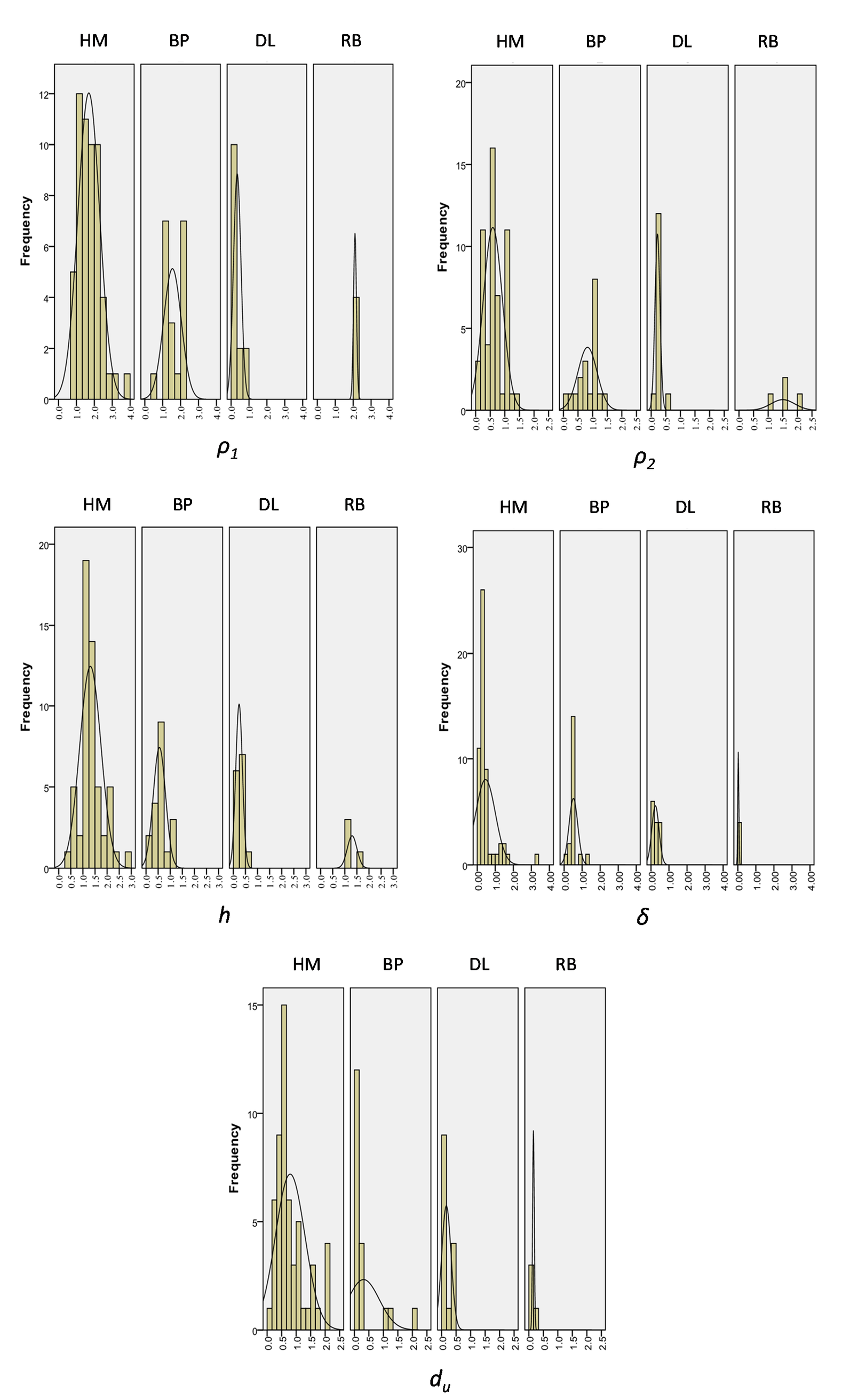

Supplement: S3 Fig — G0 was modified to mimic a change in drug dose that mimicked drug efficacies that equated to ε at 80% (black solid line), 90% (mean—dashed black line) and 95% (dotted black line) for each profile. The mean data is also shown for each profile. The limit of detection (LOD) of the viral load (400 copies/ml) is shown by the horizontal green line. (TIF) [file ppat.1006299.s003.tif]
